# The Role of Estriol and Estrone in Keratoconic Stromal Sex Hormone Receptors

**DOI:** 10.3390/ijms23020916

**Published:** 2022-01-14

**Authors:** Paulina Escandon, Sarah E. Nicholas, Rebecca L. Cunningham, David A. Murphy, Kamran M. Riaz, Dimitrios Karamichos

**Affiliations:** 1 North Texas Eye Research Institute, University of North Texas Health Science Center, Fort Worth, TX 76107, USA; paulina.escandon@unthsc.edu (P.E.); sarah.nicholas@unthsc.edu (S.E.N.); 2 Department of Pharmaceutical Sciences, University of North Texas Health Science Center, Fort Worth, TX 76107, USA; rebecca.cunningham@unthsc.edu; 3 Department of Ophthalmology, Dean McGee Eye Institute, University of Oklahoma Health Sciences Center, Oklahoma City, OK 73104, USA; david-murphy@dmei.org (D.A.M.); kamran-riaz@dmei.org (K.M.R.); 4 Department of Pharmacology and Neuroscience, University of North Texas Health Science Center, Fort Worth, TX 76107, USA

**Keywords:** keratoconus, sex hormones, cornea

## Abstract

Keratoconus (KC) is a progressive corneal thinning disease that manifests in puberty and worsens during pregnancy. KC onset and progression are attributed to diverse factors that include: environmental, genetics, and hormonal imbalances; however, the pathobiology remains elusive. This study aims to determine the role of corneal stroma sex hormone receptors in KC and their interplay with estrone (E1) and estriol (E3) using our established 3D in vitro model. Healthy cornea stromal cells (HCFs) and KC cornea stromal cells (HKCs), both male and female, were stimulated with various concentrations of E1 and E3. Significant changes were observed between cell types, as well as between males and females in the sex hormone receptors tested; androgen receptor (AR), progesterone receptor (PR), estrogen receptor alpha (ERα), and estrogen receptor beta (ERβ) using Western blot analysis. E1 and E3 stimulations in HCF females showed AR, PR, and ERβ were significantly upregulated compared to HCF males. In contrast, ERα and ERβ had significantly higher expression in HKC’s females than HKC’s males. Our data suggest that the human cornea is a sex-dependent, hormone-responsive tissue that is significantly influenced by E1 and E3. Therefore, it is plausible that E1, E3, and sex hormone receptors are involved in the KC pathobiology, warranting further investigation.

## 1. Introduction

Keratoconus (KC) is a progressive disorder in which the normally “round” cornea thins and bulges out, resulting in significant vision impairment. The complexity of KC in the context of progression, onset, and pathology is still not well understood. KC affects both males and females [1] and contributing factors include: genetics [2,3,4,5,6,7], environment (external factors) [2,4], hormonal imbalances [8,9], and physical eye rubbing [10,11,12,13,14]. Sex and age have been associated with the onset of KC, beginning in adolescence [15,16,17] and with significant exacerbation during pregnancy [16,18,19,20], coinciding with major hormonal changes.

Sex hormone changes in KC have been recently implicated in its pathogenesis [9,18,21,22]. Sex hormones are accessible to all tissues through blood circulation; however, specific receptors are required for these hormones to function and have an impact. During pregnancy, the fluctuation of sex hormones has been correlated to causing corneal alterations in curvature, central corneal thickness, and corneal volume [23,24,25]. It is believed that these hormonal changes exacerbate the progression of KC during pregnancy and can extend up to six months postpartum [18]. In addition, one study has shown that changes in estrogen levels during menopause were associated with alterations in corneal curvature in females [26].

Expression of sex hormone receptors in the human cornea has been reported; however, their role and function pertaining to corneal processes are still unclear [27,28,29,30,31]. In addition, the presence of sex hormone receptors in other ocular tissues such as lens [30,32], retina [30,33], lacrimal gland [30,34], iris [30,32], and ciliary body [30] have been reported, as well as sex hormone influence in structure and function of lacrimal glands impacting ocular diseases such as dry eye [35]. It is, therefore, critical to delineate the interplay between KC, sex hormones, and their receptors. 

Levels of estrogen-derived hormones estrone (E1) and estriol (E3), are of particular interest as they fluctuate during puberty, menstruation, pregnancy, and menopause, suggesting a possible impact on the KC pathobiology. To date, there is no evidence on whether E1 and E3 fluctuations affect corneal homeostasis in KC. Interestingly, our group has previously reported that both, E1 and E3 are downregulated in KC blood (plasma) and saliva samples compared to their healthy counterparts, independent of age, sex, or KC severity [22]. The impact of E1/E3 in KC-stromal microenvironment (i.e., in vitro) has yet to be investigated.

In the current study, we investigated the effects of E1 and E3 on sex hormone receptors present in corneal stromal cells derived from healthy and keratoconus donors, utilizing our established 3D KC self-assembled ECM in vitro model. Overall, our data reveal for the first time, the impact of E1 and E3 on sex hormone receptors present in KC corneal stromal cells and highlights their sex-dependent modulation.

## 2. Results

Sex hormone receptors AR, PR, ERα, and ERβ expression was examined using Western blot assays for both HCF and HKC constructs post-stimulation of E1 or E3 for 4 weeks. Fold change of sex hormone receptors expression to GAPDH (housekeeping) expression are demonstrated in the results. The overall sex hormone receptor expression was analyzed from two HCFs and two HKCs cornea donors, where each cornea donor was performed in triplicate, making an *n* = 6 per cell type. Data were also stratified by sex in both HCFs (one female and one male) and HKCs (one female and one male) cornea donors, with each cornea donor constructs performed in triplicate making an *n* = 3 per sex-cell type. 

### 2.1. Impact of E1 on Sex Hormone Receptors 

Protein expression was investigated for AR, PR, ERα, and ERβ in 3D HCF and HKC constructs in response to 10, 50, and 150 ng/mL E1 stimulation at four weeks. Data were also stratified by sex in both HCFs and HKCs.

#### 2.1.1. Androgen Receptor

**Figure 1:**
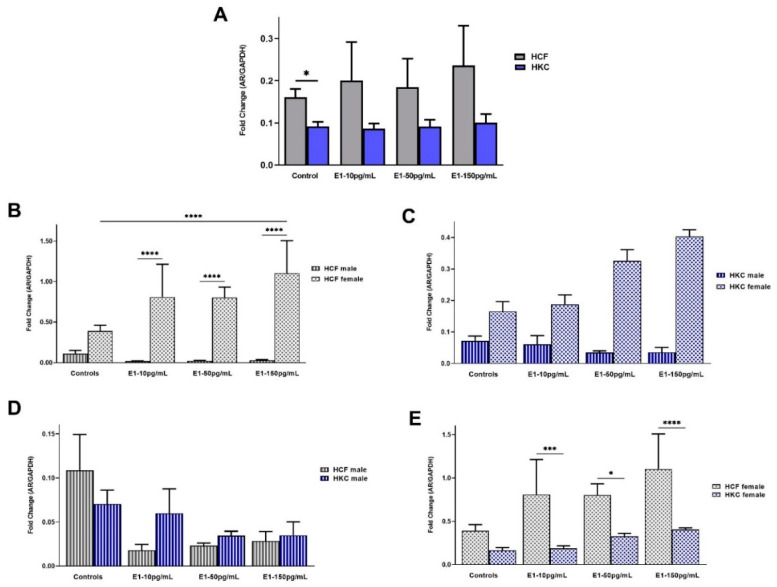
Expression of AR in 3D HCFs and HKCs in response to 10, 50, 150 pg/mL E1 stimulation for four weeks. (**A**) Overall AR expression in HCFs and HKCs (*n* = 6). (**B**) Sex-specific expression of AR in HCFs (*n* = 3). (**C**) Sex-specific AR expression in HKCs (*n* = 3). (**D**) Male-specific AR expression in HCFs vs. HKCs (*n* = 3). (**E**) Female-specific expression of AR in HCFs vs. HKCs. (*n* = 3). * *p* < 0.05, *** *p* < 0.001, and **** *p* < 0.0001.

The overall AR expression between HCFs and HKCs showed significant upregulation in HCF controls when compared to HKC controls (Figure 1A). However, E1 stimulation did not show substantial differences in AR expression between HCFs and HKCs (Figure 1A).

In terms of sex-specific AR expression in HCFs, females showed significantly higher expression than males in all E1 concentrations tested (Figure 1B). In addition, expression of AR in HCF females was upregulated significantly when stimulated with 150 pg/mL of E1 compared to HCF male controls (Figure 1B). Interestingly, sex-specific AR expression in HKCs showed no significant changes (Figure 1C). 

No statistically significant differences were observed when comparing the male-specific AR expression in HCFs or HKCs (Figure 1D). On the other hand, the female-specific AR expression was significantly downregulated in HKC females compared to HCF females under all E1 concentrations (Figure 1E).

#### 2.1.2. Progesterone Receptor

**Figure 2:**
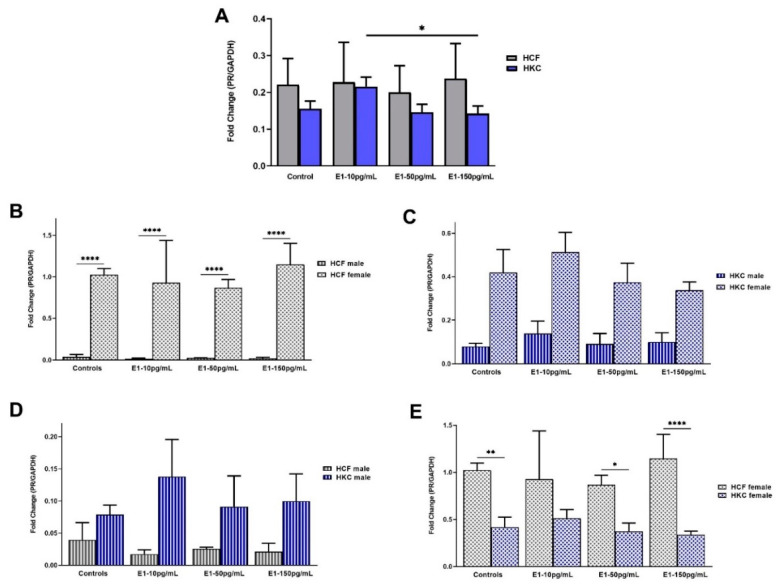
Expression of PR in 3D HCFs and HKCs in response to 10, 50, 150 pg/mL E1 stimulation for four weeks. (**A**) Overall PR expression in HCFs and HKCs (*n* = 6). (**B**) Sex-specific expression of PR in HCFs (*n* = 3). (**C**) Sex-specific PR expression in HKCs (*n* = 3). (**D**) Male-specific PR expression in HCFs vs. HKCs (*n* = 3). (**E**) Female-specific expression of PR in HCFs vs. HKCs (*n* = 3). * *p* < 0.05, ** *p* < 0.01, and **** *p* < 0.0001.

The overall PR expression was similar between HCFs and HKCs, with no significant differences. However, 150 pg/mL E1 stimulation showed significantly lower PR expression compared to 10 pg/mL E1 stimulation, in HKCs (Figure 2A).

Sex-specific PR expression in HCFs showed that females have significantly upregulated PR expression when compared to their male counterparts both in controls and all E1 concentrations tested (Figure 2B). Conversely, there were no significant differences in sex-specific PR expression in HKCs regardless of E1 stimulation (Figure 2C).

When HCF males were compared to HKC males for PR expression, no significant differences were observed (Figure 2D). However, HCF females showed significantly higher AR expression when compared to HKC females in controls and 50 pg/mL and 150 pg/mL of E1 stimulation (Figure 2E).

#### 2.1.3. Estrogen Receptor Alpha

**Figure 3:**
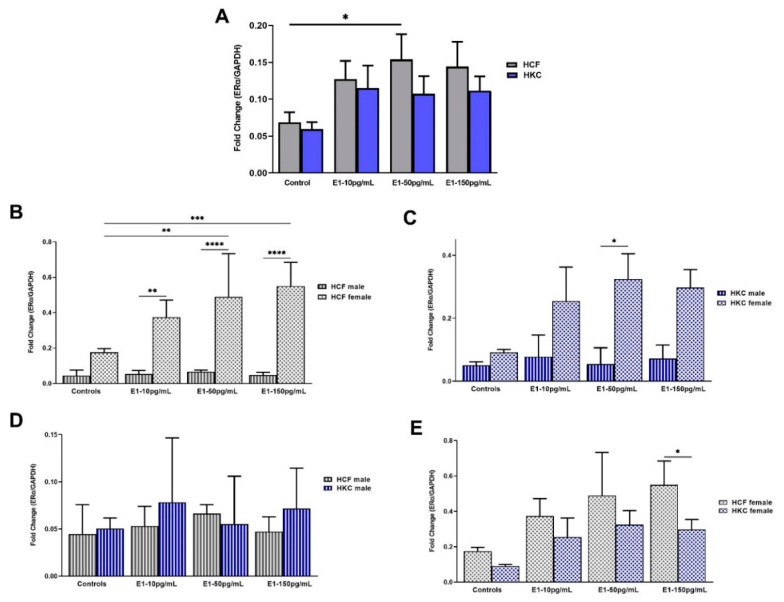
Expression of ERα in 3D HCFs and HKCs in response to 10, 50, 150 pg/mL E1 stimulation for four weeks. (**A**) Overall ERα expression in HCFs and HKCs (*n* = 6). (**B**) Sex-specific expression of ERα in HCFs (*n* = 3). (**C**) Sex-specific ERα expression in HKCs (*n* = 3). (**D**) Male-specific ERα expression in HCFs vs. HKCs (*n* = 3). (**E**) Female-specific expression of ERα in HCFs vs. HKCs. * *p* < 0.05, ** *p* < 0.01, *** *p* < 0.001, and **** *p* < 0.0001.

The overall expression of ERα in HCFs and HKCs showed no statistical differences. However, HCFs stimulated with 50 pg/mL of E1 showed significant upregulation in ERα expression when compared to HCF controls (Figure 3A). No significant differences were observed between HCFs and HKCs when stimulated with E1.

When comparing sex-specific ERα expression in HCFs, females showed significantly higher expression than males under all E1 concentrations tested (Figure 3B). In addition, the expression of ERα in HCF females was significantly upregulated with 50 pg/mL and 150 pg/mL of E1 when compared to HCF female controls. Sex-specific ERα expression in HKCs showed significant upregulation with 50 pg/mL of E1 in females compared to their male counterparts (Figure 3C). 

There were no significant differences in ERα expression when comparing HCF males with HKC males (Figure 3D). However, ERα expression in HCF females was significantly upregulated compared to HKC females, when stimulated with 150 pg/mL of E1 (Figure 3E).

#### 2.1.4. Estrogen Receptor Beta

**Figure 4:**
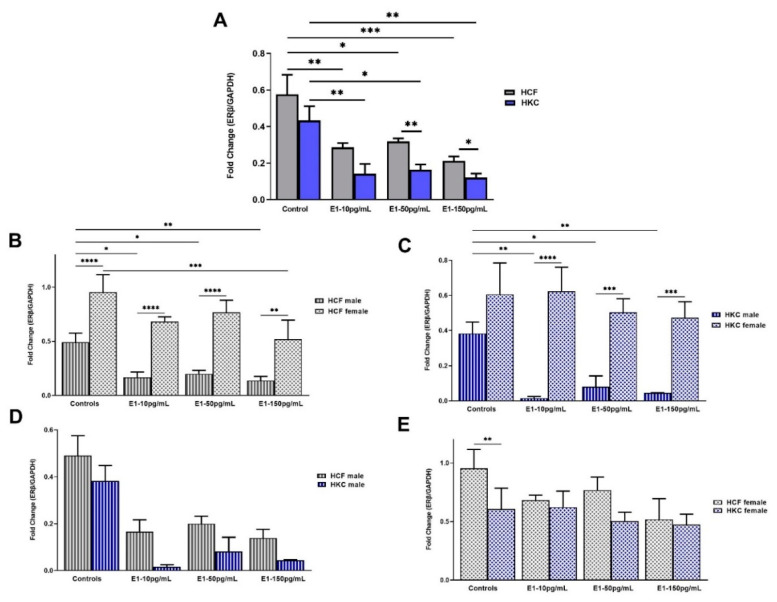
Expression of ERβ in 3D HCFs and HKCs in response to 10, 50, 150 pg/mL E1 stimulation for four weeks. (**A**) Overall ERβ expression in HCFs and HKCs (*n* = 6). (**B**) Sex-specific expression of ERβ in HCFs (*n* = 3). (**C**) Sex-specific ERβ expression in HKCs (*n* = 3). (**D**) Male-specific ERβ expression in HCFs vs. HKCs (*n* = 3). (**E**) Female-specific expression of ERβ in HCFs vs. HKCs (*n* = 3). * *p* < 0.05, ** *p* < 0.01, *** *p* < 0.001, and **** *p* < 0.0001.

The overall ERβ expression in HCFs was significantly upregulated compared to HKCs when stimulated with 50 pg/mL and 150 pg/mL of E1 (Figure 4A). In addition, HCF controls showed upregulated ERβ expression compared to HCFs under all E1 concentrations tested (Figure 4A). Similarly, ERβ expression in HKC controls was significantly upregulated compared to E1-stimulated HKCs, independent of the concentration (Figure 4A).

When comparing sex-specific ERβ expression in HCFs, females showed significant upregulation compared to males, for controls and under all E1 concentrations tested (Figure 4B). Expression of ERβ in HKC males was downregulated significantly with 10 pg/mL, 50 pg/mL, and 150 pg/mL of E1, compared to HKC male controls. We also found that HCF females’ ERβ expression was downregulated with 150 pg/mL E1 stimulation compared to HKC female controls. Regarding sex-specific ERβ expression in HKCs, ERβ expression was significantly upregulated in females compared to males under all E1 concentrations (Figure 4C). Interestingly, ERβ expression was significantly downregulated in HKC males under all E1 concentrations compared to controls.

When HCF males were compared to HKC males for ERβ expression, no significant differences were identified (Figure 4D). In addition, E1 stimulation did not show significant differences in ERβ expression between HCFs males and HKCs males (Figure 4D). On the other hand, ERβ expression was significantly upregulated in HCF female controls than HKC female controls (Figure 4E).

### 2.2. Impact of E3 on Sex Hormone Receptors

Protein expression was investigated for AR, PR, ERα, and ERβ in 3D HCFs and HKCs constructs at four weeks with or without E3 stimulation (2, 15, and 30 ng/mL). Findings were also stratified by sex in both HCFs and HKCs.

#### 2.2.1. Androgen Receptor

**Figure 5:**
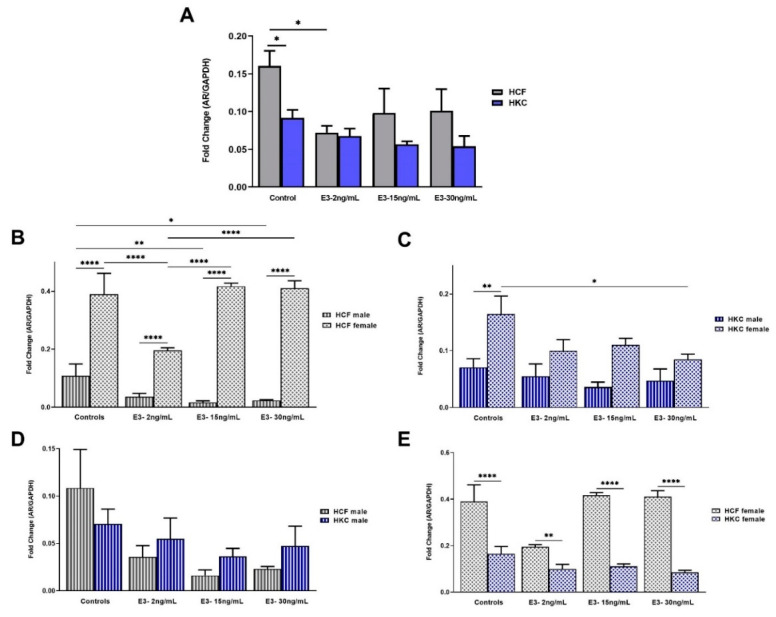
Expression of AR in 3D HCFs and HKCs in response to 2, 15, 30 ng/mL E3 stimulation for four weeks. (**A**) Overall AR expression in HKCs and HKCs (*n* = 6). (**B**) Sex-specific expression of AR in HCFs (*n* = 3). (**C**) Sex-specific AR expression in HKCs (*n* = 3). (**D**) Male-specific AR expression in HCFs vs. HKCs (*n* = 3). (**E**) Female-specific expression of AR in HCFs vs. HKCs. * *p* < 0.05, ** *p* < 0.01, and **** *p* < 0.0001.

When comparing overall AR expression between HCFs and HKCs, HCF controls showed significant upregulation compared to HKC controls (Figure 5A). In addition, 2 ng/mL E3 stimulation led to significant AR downregulation in HCFs compared to controls. Furthermore, AR expression in HKCs was unaffected by E3 stimulation (Figure 5A). 

In terms of sex-specific AR expression in HCFs, females showed significantly upregulated expression compared to males in all E3 concentrations tested (Figure 5B). The expression of AR in HCF males was downregulated significantly with 15 ng/mL and 30 ng/mL of E3, compared to HCF male controls. Contrastingly, HCF females showed downregulated AR expression with 2 ng/mL, but no changes with 15 ng/mL or 30 ng/mL E3 stimulation. HCF females stimulated with 2 ng/mL of E3 have the lowest AR expression compared to the other conditions, including controls (Figure 5B). Regarding sex-specific AR expression in HKCs, male controls showed significantly downregulated expression than female controls (Figure 5C). Interestingly, AR expression in HKC males was independent of E3 stimulation; whereas, in HKC females, it was only modulated at 30 ng/mL.

Intriguingly, when comparing HCF and HKC males for AR expression, no significant differences were identified (Figure 5D). However, AR expression was significantly downregulated in HKC females compared to HCF females in controls and under all E3 concentrations tested (Figure 5E).

#### 2.2.2. Progesterone Receptor

**Figure 6:**
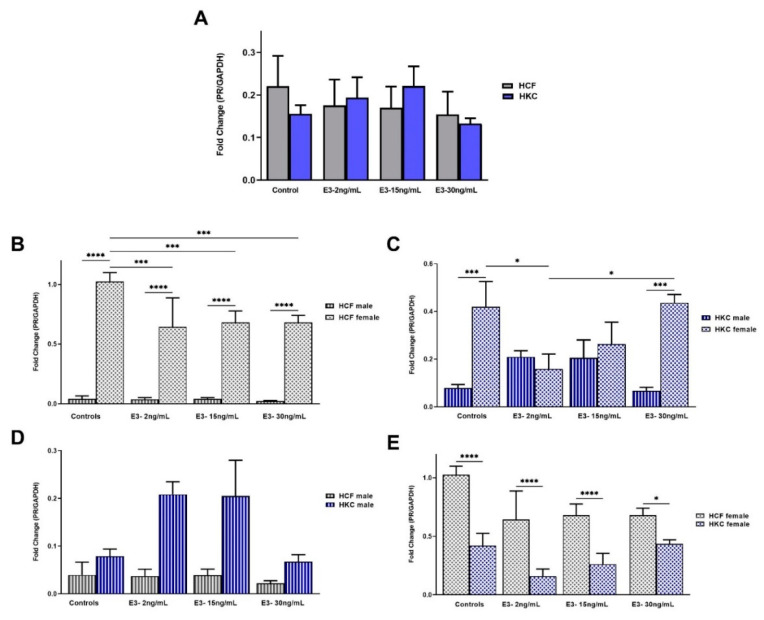
Expression of PR in 3D HCFs and HKCs in response to 2, 15, 30 ng/mL E3 stimulation for four weeks. (**A**) Overall PR expression in HCFs and HKCs (*n* = 6). (**B**) Sex-specific expression of PR in HCFs (*n* = 3) (**C**) Sex-specific PR expression in HKCs. (**D**) Male-specific PR expression in HCFs vs. HKCs. (**E**) Female-specific expression of PR in HCFs vs. HKCs. * *p*< 0.05, *** *p* < 0.001, and **** *p* < 0.0001.

The overall PR expression between HCFs and HKCs showed no significant differences under all E3 concentrations tested or controls (Figure 6A). 

There are substantial differences in sex-specific PR expression in HCFs, where females showed significantly upregulated expression compared to males in controls and under all E3 concentrations tested (Figure 6B). In addition, expression of PR was significantly downregulated for HCF females in all E3 concentrations compared to HCF female controls. In terms of sex-specific PR expression in HKCs, male controls and 30 ng/mL E3 showed significant downregulation when compared to their female counterparts (Figure 6C). In addition, a 2 ng/mL E3 stimulation led to significant PR downregulation in HKC females compared to controls and 30 ng/mL E3. 

No significant differences in PR expression were observed when comparing HCF and HKC males, regardless of E3 concentrations tested (Figure 6D). On the other hand, HCF females showed significant upregulation of PR expression compared to HKC females in controls and all E3 concentrations tested (Figure 6E).

#### 2.2.3. Estrogen Receptor Alpha

**Figure 7:**
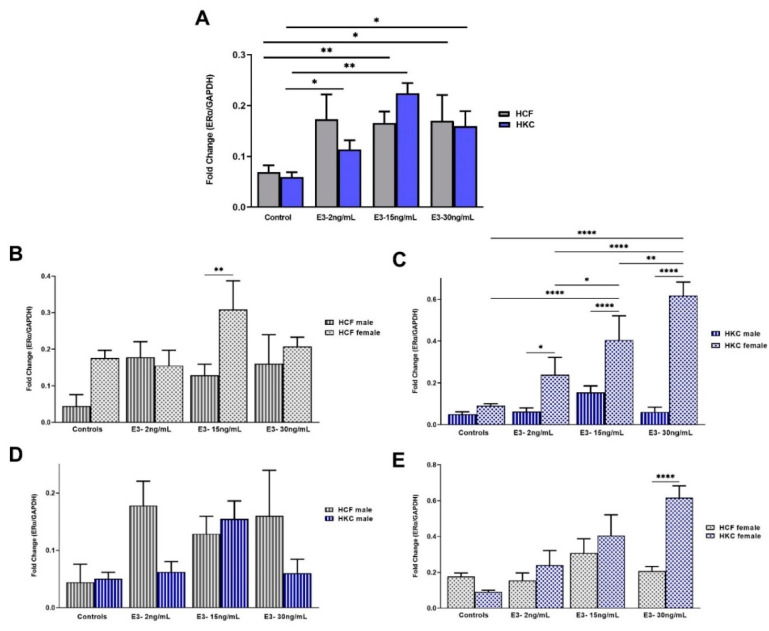
Expression of ERα in 3D HCFs and HKCs in response to 2, 15, 30 ng/mL E3 stimulation for four weeks. (**A**) Overall ERα expression in HCFs and HKCs (*n* = 6). (**B**) Sex-specific expression of ERα in HCFs (*n* = 3). (**C**) Sex-specific ERα expression in HKCs (*n* = 3). (**D**) Male-specific ERα expression in HCFs vs. HKCs (*n* = 3). (**E**) Female-specific expression of ERα in HCFs vs. HKCs (*n* = 3). * *p* < 0.05, ** *p* < 0.01, and **** *p* < 0.0001.

The overall ERα expression showed significant upregulation in HCFs stimulated with 15 ng/mL and 30 ng/mL of E3 when compared to controls (Figure 7A). In addition, HKCs showed significant upregulation of ERα when stimulated with all concentrations of E3 compared to controls.

When comparing sex-specific ERα expression in HCFs, males showed significantly downregulated expression than females, when stimulated with 15 ng/mL of E3 (Figure 7B). In terms of sex-specific ERα expression in HKCs, females showed significant upregulation compared to males under all E3 concentrations (Figure 7C). HKC female controls and stimulated with 2 ng/mL of E3 showed significant ERα downregulation compared to 15 ng/mL and 30 ng/mL E3s. In addition, 15 ng/mL E3 led to significant ERα downregulation compared to 30 ng/mL E3 in HKC females. 

When comparing HCF to HKC males for ERα expression, no significant differences were found, regardless of E3 stimulation (Figure 7D). Contrastingly, a 30 ng/mL E3 stimulation led to significant ERα downregulation in HCF females compared to HKC females (Figure 7E).

#### 2.2.4. Estrogen Receptor Beta

**Figure 8:**
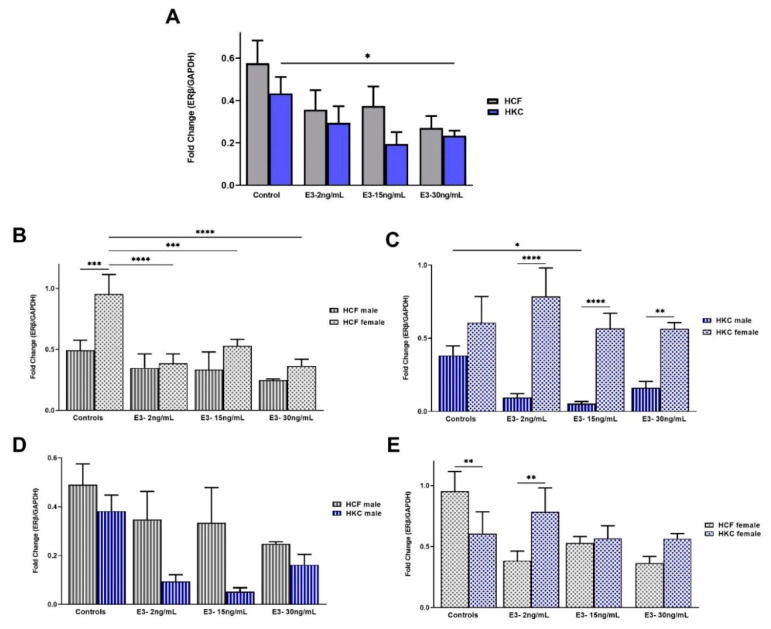
Expression of ERβ in 3D HCFs and HKCs in response to 2, 15, 30 ng/mL E3 stimulation for four weeks. (**A**) Overall ERβ expression in HCFs and HKCs (*n* = 6). (**B**) Sex-specific expression of ERβ in HCFs (*n* = 3). (**C**) Sex-specific ERβ expression in HKCs (*n* = 3). (**D**) Male-specific ERβ expression in HCFs vs. HKCs (*n* = 3). (**E**) Female-specific expression of ERβ in HCFs vs. HKCs (*n* = 3). * *p* < 0.05, ** *p* < 0.01, *** *p* < 0.001, and **** *p* < 0.0001.

The overall ERβ expression showed no significant differences between HCFs and HKCs (Figure 8A). However, HKC controls showed significant downregulation of ERβ expression compared to HKCs stimulated with 30 ng/mL of E3. 

Regarding sex-specific ERβ expression in HCFs, male controls showed significantly lower expression than female controls (Figure 8B). In addition, HCF females under all E3 concentrations showed significant ERβ downregulation compared to controls. In terms of sex-specific ERβ expression findings in HKCs, males showed downregulated ERβ expression following 15 ng/mL E3 stimulation when compared to male controls (Figure 8C). In addition, HKC females showed significant ERβ upregulation compared to HKC males, in all E3 concentrations tested.

When comparing HCF and HKC males for ERβ expression, no significant differences were noted (Figure 8D). However, HCF female controls showed significant upregulation of ERβ compared to HKC females (Figure 8E). ERβ expression in HCF females with 2 ng/mL of E3 stimulation showed significant downregulation compared to HKC counterparts.

## 3. Discussion

KC presents during puberty [15,30] as hormone levels considerably change and its progression stabilizes during menopause/andropause, as another oscillation of hormone levels happens in life [36]. Hence, hormonal changes significantly contribute to the variations by age and sex in KC occurrences. In addition, KC progression in pregnancy supports the relationship between hormone changes and the human cornea [16,18,19,20]. Therefore, we advance on a pathogenesis theory that KC’s fluctuating progression during puberty, pregnancy, and menopause is strongly influenced by the effects of hormone levels and their receptors present in the cornea. 

In the current study, we investigated the effects of two hormones, E1 and E3, on sex hormone receptors present in the human cornea. The results are summarized in Table 1. Our study reveals that sex hormone receptors in the cornea are impacted by hormone stimulation (E1 and E3), and their expression is sex-dependent. To date, no cellular/molecular studies have demonstrated the effects of sex hormones in the human cornea and whether this dynamic differs between heathy and KCs. Interestingly, studies have shown that estrogen modifies corneal biomechanics in pig corneas [37,38], yet there is no such evidence in human corneas. An animal model studying keratoconus has also shown hormone (androgen) dependency, where corneal changes occurred exclusively in male mice, and when castrated the changes were drastically diminished [39]. In addition, when female mice were simulated with androgen, they showed a similar corneal phenotype as male mice [39].

E1 and E3 are estrogens produced by the ovaries in females and by the testes in males. Throughout menopause, E1 levels are predominant in the circulation and are responsible for sexual development, function, and can serve as a repository for estrogen (converting E1 to estrogen) [40,41]. A recent in vitro study showed that estrogen receptors mediated E1 biomechanical action in bone tissue to promote osteoblasts differentiation, which can aid post-menstrual women with osteoporosis [42]. In addition, clinical studies have shown E3 significantly improves bone mineral density in women with post-menopausal osteoporosis [43]. Clinical studies have also shown E3 effectiveness in treating symptomatic vaginal atrophy and abnormal vaginal flora therapy in pre- and post-menopausal women [44]. 

The physiologic process that happens during menopause has been shown to alter corneal topography in females [26]. Studies have shown that hormone replacement therapy on post-menopausal patients with KC could lead to the progression of KC [45,46]. Our study showed that E1 stimulation activated AR and ERα expression in HCF females compared to HCF males and HKC females (Figure 1B,E). In addition, our results showed E1 stimulation affected AR, ERα, and ERβ, with little to no response from PR expression in HCF females A study by Bjerregaard-Olesen has also shown that ER and AR can transactivate by cell culture exposure to sulfated E1, dependent on concentration [47]. Our results not only show similar activation of the receptors for HCF response to E1, but also reveal that the response is female-dependent for AR, ERα, and ERβ. It is plausible that KC, in males and females, is altered depending on which sex hormone receptor is modulated. 

**Figure 9:**
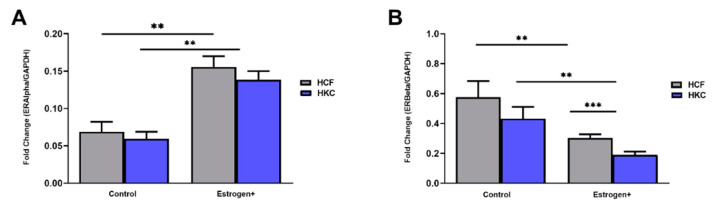
Expression of ERα and ERβ in 3D HCFs and HKCs in response to estrogen+ (E1 and E3) stimulation for four weeks. (**A**) Overall ERα expression in HCFs and HKCs (*n* = 6). (**B**) Overall expression of ERβ in HCFs and HKCs (*n* = 6). ** *p* < 0.01, and *** *p* < 0.001.

E3 levels increase progressively throughout pregnancy [48]; thus, there may be a direct association of E3 levels with disease exacerbation in KC. A recent study showed that in vitro fertilization (ivf) treatment increased estrogen levels, leading to the progression of KC in three patients [49]. Our results showed differences in AR and PR expression modulated by E3 between HCF and HKC females (Figure 5A and Figure 6E). In addition, for HKCs, there was an inverse correlation between E3 modulation overall expression of ERα (increased expression with E3) and ERβ (decreased expression with E3) (Figure 7A and Figure 8A). In addition, we analyzed the impact of both estrogen stimulations on the overall expression of ERα and ERβ (Figure 9A,B). The combined estrogen stimulations demonstrated an inverse response between ERα and ERβ compared to controls for both HCF’s and HKC’s. However, further studies must be performed using an E1 and E3 combination stimulation to identify if the results are actually due to estrogen stimulation.

It is clear that HKC’s sex hormone receptors respond to sex hormones, which may correlate with the response in KC’s fluctuation in corneal changes observed during puberty, menstrual cycle, pregnancy, and menopause. Previous studies have also reported corneal thickness alterations in different menstrual cycle stages [50,51,52]. These changes can be attributed to hormone influence on hormonal receptors, significantly causing corneal thickness alterations. Our study revealed E1 and E3 modulation on hormone receptors present in human corneal stromal cells. Although we did not observe morphological changes in the cells from E1 and E3 stimulation, in the future, studies on cellular proliferation and toxicity are needed to rule out any adverse effects from all concentrations tested.

Sex hormone receptors, including AR, PR, ERα, and ERβ, have not only been identified in the human cornea [27,53] and lacrimal gland [34,35,54] but are also implicated in ocular diseases such as dry eye [55]. A previous study showed that PR expression was inhibited, whereas AR was activated in KC corneas [28]. Additionally, ERα and ERβ showed similar expression levels in control and KC corneas [28]. Our present study found that E1 and E3 modulated the activation of AR, PR, ERα, and ERβ in HCF females compared to HCF males. (Figure 1B, Figure 2B, Figure 3B, Figure 4B, Figure 5B, Figure 6B, Figure 7B, Figure 8B). Although AR is associated with male reproduction, AR is also essential for female fertility, specifically for ovarian follicle development and full functionality during ovulation [56]. Therefore, AR, PR, ERα, and ERβ, found in human corneas play a role in corneal physiology and may be influenced by any hormones found in fluids surrounding the eye, such as tear fluid. While we investigated the E1 and E3 response in cornea stromal cells from healthy and KC donors, sex hormone receptors have also been found in the nuclei of corneal epithelial and endothelial cells [27]. It is, therefore, critical that future studies investigate the response, if any, of sex hormone receptors in cornea epithelial and endothelial cells with stimulation of E1 and E3. Such studies will provide a complete picture of the impact of these receptors, in KC.

Additional studies will be needed, including additional donors per sample group in order to delineate the sex differences observed. KC is a diverse, complicated, and multifactorial disease of the human cornea. Our in vitro studies highlight the importance of sex bias in KC. The topic is certainly worthy of more attention and research studies, given that clinical cases support such a motion. The major limitation of our study is the low number of donor samples, tested per group. Therefore, it is currently unknown how our finding might change in the future when factors such as age, or KC severity are considered. Regardless, our studies are novel and should stimulate further discussions, and scientific discoveries in the field. 

Our data showed that hormonal receptors are responsive to E1 and E3; however, the downstream signaling mechanism(s) behind the activation/deactivation and impact of such activity remain unknown. A rather obvious theory/hypothesis would be that E1 and E3 bind to the hormone receptors on cell membranes, thereby causing a signaling cascade that leads to the activation of these receptors. Nonetheless, further research is warranted to investigate if activation/deactivation of sex hormone receptors modulates the human corneal microenvironment. Our findings are novel and support existing clinical reports and case studies on the importance of hormones in KC pathobiology, emphasizing the presence and role of sex hormone receptors in the human cornea. Further studies are necessary to determine how these estrogen-derived hormones can be utilized as therapeutics for KC progression and development. 

## 4. Materials and Methods

### 4.1. Ethical Approval

The study adhered to the tenets of the Declaration of Helsinki and was performed with the North Texas Regional Institutional Review Board (IRB) approval (protocol #2020-031). Written and informed consent was obtained prior to tissue collection, all methods were performed according to federal and institutional guidelines and all human samples were de-identified before analysis. A total of four donor tissues were used including; a healthy female (72 y/o), a healthy male (64 y/o), a KC female (43 y/o), and a KC male (19 y/o).

### 4.2. Corneal Stromal Cells Isolation

Human corneal stromal cells were isolated from both male and female KC (HKC) and healthy (HCF) corneas. Briefly, corneas were suspended in sterile PBS (Thermo Fisher Scientific, Waltham, MA, USA) to maintain hydration. Using a scalpel blade, one-third of the cornea was removed, saved for protein extraction, and used for future research studies. The rest of the cornea was then scraped using a razor for approximately 10 s from top and bottom to remove the cornea epithelium and endothelium. The corneal stromal explants were then rinsed with sterile PBS and cut into 2 × 2 mm pieces. The explant pieces were adhered to a T25 flask by incubation for 45 min at 37 °C in 5% CO_2_. Finally, corneal stromal explants were incubated with 10% fetal bovine serum (FBS) (Atlanta Biologicals, Flowery Branch, GA, USA), and 1% antibiotic/antimycotic (AA) (Life Technologies, Grand Island, NY, USA) in Eagle’s Minimum Essential Media (EMEM) (American Type Culture Collection, Manassas, VA, USA) for one week. After the first week, the media was changed every two days for four weeks. Once cells reached ~80% confluency, they were isolated with trypsin and frozen with cryogenic protection for further processing.

### 4.3. Cell Cultures and Stimulations

Male and female HCFs and HKCs were cultured in T175 flasks until ~80% confluency. Both cell types were counted following trypsinization and sub-cultured at a density of 1 × 10^6^ cells/well in 6-well 3D (transwell) plates (VWR, Radnor, PA, USA) with 10% FBS, 1% AA in EMEM. Control constructs were stimulated with EMEM containing 10% FBS, 1% AA, and 0.5 mM stable vitamin C (0.5 mM 2-O-α-D-glucopyranosyl-L-ascorbic acid, Sigma-Aldrich, St. Louis, MO, USA), as previously reported [57,58,59,60]. Constructs were also treated with E1 and E3 at three concentrations each as follows: E1—10 pg/mL, 50 pg/mL, and 150 pg/mL (E1253, Sigma-Aldrich, St. Louis, MO, USA) and E3—2 ng/mL, 15 ng/mL and 30 ng/mL (E9750, Sigma-Aldrich, St. Louis, MO, USA) in EMEM containing 10% FBS, 1% AA, and 0.5 mM stable vitamin C. All constructs were cultured for four weeks before further analysis.

### 4.4. Protein Extraction and Quantification

Protein was extracted from each construct using 100 µL of 1X radioimmunoprecipitation assay buffer and a protease inhibitor lysis cocktail (RIPA, Sigma-Aldrich, St. Louis, MO, USA). In brief, 100 µL of lysis cocktail was added to each tube and kept on ice throughout the procedure. Media was then aspirated and discarded, followed by a 1mL cold PBS wash, twice for each construct. All samples were incubated in an ice bucket at 4 °C for 30 min in the lysis cocktail. The cell lysates were centrifuged at 4 °C for 15 min at 12,000 rpm, and the supernatant was carefully collected into a new tube for Western blot analysis.

Protein samples were quantified using Pierce™ BCA Protein Assay Kit (Thermo Fisher Scientific, Waltham, MA USA). In brief, 10 µL of Pierce™ Bovine Serum Albumin Standards (Thermo Fisher Scientific, Waltham, MA, USA) were added to a Corning™ Costar™ 96-well-plate (Thermo Fisher Scientific, Waltham, MA, USA). Samples were then vortexed and 10 µL were added to the 96-well plate. Finally, 200 µL of Pierce™ BCA reagent A and reagent B working solution was added to each well, mixed with shaker for 30 s, and incubated at 37 °C with 5.0% CO_2_ for 30 min. After incubation, absorbance was measured using the BioTek EPOCH2 microplate reader (BioTek, Winooski, VT, USA) set at 562 nm. BioTek software was used to calculate results by plotting the absorbance values using linear regression to the standards.

### 4.5. Western Blot Assay

Normalized protein samples were denatured, added into Novex 4–20% Tris-glycine mini Wedge 12-well gels (Invitrogen, ThermoFisher Scientific, Waltham, MA, USA), then run via electrophoresis at 225 volts for 30-min and transferred into nitrocellulose membranes using the Invitrogen iBlot 2 Dry Blotting system at setting P0. 

The membranes were then blocked in 5% milk with Trans buffered saline with tween 20 (TBST) and incubated at room temperature on a shaker for one hour. Next, the membranes were incubated overnight on a rocker at 4 °C in the following rabbit polyclonal antibodies: AR (ab133273, Abcam, Cambridge, MA, USA), PR (ab191138, Abcam, Cambridge, MA, USA), ERα (ab75635, Abcam, Cambridge, MA, USA), and ERβ (ab3576, Abcam, Cambridge, MA, USA) at a 1:250 dilution or mouse monoclonal GAPDH antibody conjugated with AlexaFluor 680 at a 1:2000 dilution (ab184095, Abcam, Cambridge, MA, USA). The membranes were washed three times for five minutes with TBST and incubated with donkey anti-rabbit IgG (H+L) AlexaFluor 568 antibody (ab175470, Abcam, Cambridge, MA, USA) at a 1:2000 dilution for 1 h on a rocker at room temperature. After washing, all membranes were imaged using the iBright FL 15000 imaging system (ThermoFisher Scientific, Waltham, MA, USA). Results were analyzed using iBright analysis software (ThermoFisher Scientific, Waltham, MA, USA) and results were normalized to the expression of GAPDH housekeeping.

### 4.6. Statistical Analysis

GraphPad Prism 7.0 was used for performing all statistical analyses (GraphPad Software, Inc., La Jolla, CA, USA). Data were analyzed using one-way analysis of variance (ANOVA) and *t*-test where necessary, a value of *p* < 0.05 was considered statistically significant.

## Figures and Tables

**Table 1:** Summary for protein expression results of sex hormone receptors in of HCFs and HKCs stimulated with E1 and E3. ↑ = upregulation and ↓ = downregulation.

	E1	E3
***AR***	Overall ↑ in HCFs compared to HKCs in controls (Figure 1A). ↑ in HCF females compared to HCF males in all concentrations (Figure 1B). ↓ in HCF females controls compared to HCF females stimulated with 150 pg/mL (Figure 1B). ↑ in HCF females compared to HKC females in all concentrations (Figure 1E).	Overall ↑ in HCFs compared to HKCs in controls (Figure 5A). Overall ↓ of HCF controls compared to HCFs stimulated with 2 ng/mL (Figure 5A). ↑ in HCF females compared to HCF males in all concentrations (Figure 5B). ↑ in HCF females compared to HCF males in controls (Figure 5B). ↑ in HCF male control compared to HCF male stimulated with 15 ng/mL and 30 ng/mL (Figure 5B). ↓ in HCF females stimulated with 2 ng/mL compared to controls, 15 ng/mL, and 30 ng/mL (Figure 5B). ↑ in HKC females controls compared to HKC male controls and HKC female stimulated with 30 ng/mL (Figure 5C). ↑ in HCF females compared to HKC females in all concentrations (Figure 5E). ↑ in HCF females compared to HKC females in controls (Figure 5E).
***PR***	Overall ↑ in HKCs stimulated with 10 pg/mL compared to HKCs stimulated with150 pg/mL (Figure 2A). ↑ in HCF females compared to HCF males in controls and all concentrations tested (Figure 2B). ↑ in HCF females compared to HKC females controls in the two highest concentrations (Figure 2E).	↑ in HCF females compared to HCF males in controls and all concentrations tested (Figure 6B). ↓ in HCF females stimulated with all concentrations compared to HCF female controls (Figure 6B). ↑ in HKC female controls and stimulated with 30 ng/mL compared to HKC male controls and simulated with 30 ng/mL (Figure 6C). ↓ in HKC females stimulated with 2 ng/mL compared to HKC female controls and stimulated with 30 ng/mL (Figure 6C). ↑ in HCF females compared to HKC females controls and in the two highest concentrations (Figure 6E). ↑ in HCF females compared to HKC females stimulated with 2 ng/mL (Figure 6E).
***ERα***	Overall ↓ in HCF controls compared to HCF stimulated with 50 pg/mL (Figure 3A). ↑ in HCF females compared to HCF males stimulated with 50 pg/mL (Figure 3B). ↑ in HCF females compared to HCF males stimulated with 10 and 150 pg/mL (Figure 3B). ↓ in HCF females controls compared to HCF males stimulated at 50 and 150 pg/mL (Figure 3B). ↑ in HKC females compared to HKC males stimulated at 50 pg/mL (Figure 3C). ↑ in HCF females compared to HKC females stimulated at 150 pg/mL (Figure 3E).	Overall ↓ in HKC controls compared to HKC stimulated in all concentrations tested (Figure 7A). Overall ↓ in HCF controls compared to HCFs stimulated with 15 ng/mL and 30 ng/mL (Figure 7A). ↑ in HCF females compared to HCF males stimulated with 15 ng/mL (Figure 7B). ↑ in HKC females compared to HKC males stimulated at 15 ng/mL (Figure 7C). ↓ in HKC female controls and stimulated with 2 ng/mL compared to HKC females stimulated with 15 and 30 ng/mL (Figure 7C). ↓ in HKC males compared to HKC females in all concentrations tested (Figure 7C). ↓ in HCF females compared to HKC females stimulated with 30 ng/mL (Figure 7E).
***ERβ***	Overall ↑ in HCF controls compared to HCF in all concentrations tested (Figure 4A). Overall ↑ in HKC controls compared to HKC in all concentration tested (Figure 4A). Overall ↑ in HCF compared to HKC stimulated with 50 pg/mL (Figure 4A). Overall ↑ in HCF compared to HKC stimulated with 150 pg/mL (Figure 4A). ↑ in HCF females compared to HCF males in controls (Figure 4B). ↑ in HCF male controls compared to HCF males in all concentrations tested (Figure 4B). ↑ in HCF female control compared to HCF female stimulated with 150 pg/mL (Figure 4B). ↑ in HCF female compared to HCF male in controls and all concentration tested (Figure 4B). ↑ in HKC females compared to HKC males in all concentrations tested (Figure 4C). ↑ HKC male controls compared to HKC males in all concentrations tested (Figure 4C). ↑ in HCF female controls compared to HKC female controls (Figure 4E).	Overall ↑ in HKC controls compared to HKC stimulated with 30 ng/mL (Figure 8A). ↑ in HCF females compared to HCF males in controls (Figure 8B). ↑ in HKC male compared to HKC male stimulated with 15 ng/mL (Figure 8B). ↑ in HKC females compared to HKC males in all concentrations tested (Figure 8C). ↑ in HKC male compared to HKC male stimulated with 15 ng/mL (Figure 8C). ↑ in HCF female controls compared to HKC female controls (Figure 8E). ↑ HKC females compared to HCF females stimulated with 2 ng/mL (Figure 8E).

## Data Availability

The data presented in this study are available on request from the corresponding author.
